# Creating Short-Term Volume Flexibility in Healthcare Capacity Management

**DOI:** 10.3390/ijerph17228514

**Published:** 2020-11-17

**Authors:** Carina Fagefors, Björn Lantz, Peter Rosén

**Affiliations:** 1 Department of Pediatric Anesthesiology, Intensive Care and Neonatology, Sahlgrenska University Hospital, 413 45 Gothenburg, Sweden; carina.fagefors@vgregion.se; 2 Technology Management and Economics, Chalmers University of Technology, 412 96 Gothenburg, Sweden; 3 Department of Engineering Science, University West, 461 86 Trollhättan, Sweden; 4 Department of Business Administration, University of Gothenburg, Box 100, 405 30 Gothenburg, Sweden; peter.rosen@handels.gu.se

**Keywords:** healthcare management, volume flexibility, capacity planning, Swedish healthcare

## Abstract

It is well-known that unpredictable variations in supply and demand of capacity in healthcare systems create the need for flexibility. The main tools used to create short-term volume flexibility in the healthcare system include overtime, temporary staff from internal calling lists, moving staff across units, internal staffing pools, external staffing agencies, queuing patients, and purchasing care from external providers. We study the creation of short-term volume flexibility in healthcare systems to manage short-term capacity losses and demand fluctuations. A questionnaire was developed and distributed among healthcare managers in the Region Västra Götaland healthcare system. Respondents were asked to what extent they used each tool to create short-term flexibility in capacity. Data were analyzed using multiple regression analysis. Several significant tendencies were found, including that acute units use overtime and internal staffing pools to a larger extent, and queuing patients and external providers to a lesser extent than planned units. The prerequisites and required managerial approaches used to efficiently manage aggregate capacity in the system differ substantially between different parts of the system. These differences must be addressed when, for example, capacity pools are considered. These results serve as a stepping stone towards a more thorough understanding of efficient capacity management in healthcare systems.

## 1. Introduction

The pressure on the Swedish healthcare system has increased in recent years. International comparison shows that the quality of Swedish healthcare is sufficient, but that the waiting times for both primary and specialized care are adversely long [1,2]. Reports issued by governmental authorities and the media frequently show that the capacity to meet healthcare demand is insufficient, and that the challenges to do so are growing each year [2,3,4,5].

Ensuring that operations can meet current and future demand effectively is a fundamental task of operations managers [6]. This task represents a significant challenge for the healthcare sector because of resource scarcity, and makes it necessary to increase the utilization and efficiency of existing resources by improving the system design and overcoming inefficiencies in present processes [7,8,9,10,11]. It is important to understand the impact of variations in demand and capacity on the healthcare system [12], and to introduce tools that create flexibility in the system to better manage these variations [9,10,13]. In this paper, we define flexibility as planned responses to anticipated contingencies [14].

According to Powers and Jack [15], responding to variations is especially difficult in healthcare organizations because of the requirements of having a complex network of facilities, equipment, a trained workforce, and the fact that healthcare services have a high level of heterogeneity. Several specialty departments share resources, which makes planning even more complex [16,17] and requires resource coordination at the hospital level [18,19]. Short-term variation in healthcare capacity is caused by, for example, sick leave, temporary leave, and vacancies, while fluctuations in demand have several explanations [20,21]. From a short-term perspective, managing these variations is challenging but crucial for both controlling capacity-related costs and achieving the best possible outcome for patients.

In general, short-term flexibility refers to the ability to adapt to changing conditions using existing resources [22]. Short-term volume flexibility relates to the ability of an organization to manage output levels to respond rapidly to short-term variations in supply and demand [13,23]. The allocation phase, often referred to as rescheduling or rerostering, deals with the real-time adjustment of personnel to accommodate demand fluctuations and unplanned staff absences. Different sources of short-term volume flexibility are commonly used to manage short-term fluctuations in day-to-day (or hour-to-hour) operations, such as filling in gaps in staff schedules due to sick leave, temporary leave, vacancies, and staffing turnover [20,21]. The use of external capacity pools (i.e., temporary agency staff) is one example of a tool that is used widely to create schedule flexibility in the healthcare sector [9,10,14,24,25,26,27,28,29]. However, the use of external pools in healthcare has turned out to be a costly solution. Agency staff can be replaced with less costly internal staffing agencies to reduce the cost of temporary agency staff and to maintain the flexibility of such capacity pools in staff scheduling [25,30,31,32,33,34].

Research on volume flexibility strategies in the manufacturing sector is comprehensive; however, this is not the case in the service sector, especially in healthcare [11,13,15,35,36,37,38,39,40,41]. In this paper, we study the creation of short-term volume flexibility in healthcare systems to manage short-term capacity losses due to unplanned absenteeism and occasions of unexpected high demand. The study is focused on volume flexibility during normal circumstances, and therefore, does not consider periods of shock or great pressure on the healthcare system due to, for example, pandemics. Different tools for volume flexibility in healthcare capacity management have varying effects on, for example, cost, work environment, and quality of care. Therefore, the results have implications for future research on how capacity pools can be organized and implemented for different healthcare departments to increase operational flexibility and use resources more efficiently. There is limited research on the use and effects of a capacity pooling approach in healthcare systems, and available studies are mainly anecdotal and directed towards pools of nurses [31,33,42,43]. Therefore, the results from this paper will contribute towards a more thorough understanding of how efficient capacity management can be achieved in healthcare systems. To our knowledge, an empirical study of this kind has not been conducted from a healthcare system perspective before. 

The remainder of this paper is organized as follows. Section 2 contains a literature review of the tools used for short-term volume flexibility in healthcare systems. In Section 3, we present the empirical setting and research methodology. We present the empirical findings and discuss the results in Section 4 and Section 5. Finally, also in Section 5, we present our conclusions and recommendations for future research. 

## 2. Literature Review 

According to case studies in the literature, the tools used frequently to create short-term volume flexibility in manufacturing industries can be classified into four categories [14,22,38,44,45]: inventory buffers; slack capacity buffers, e.g., plants that operate at less than full production capacity; workforce flexibility, e.g., overtime, on-call temporary staff, cross-trained staff, staff pools, and temporary employees; and subcontracting services, e.g., during peak demand.

Each tool has different capabilities, availability constraints, and costs (e.g., overtime payment and costs of temporary staff and productivity losses). For example, inventories are not an option in service industries, as capacity cannot be stored, and, according to Jack and Powers [45], slack capacity is often not an option for healthcare managers because healthcare organizations cannot hold capacity beyond the average level of demand due to limited financial resources or recruitment problems. Furthermore, healthcare organizations are usually unable to charge higher fees to cover increasing costs to maintain slack capacity [13,46]. When slack capacity is not an option for creating short-time volume flexibility, different cross-training policies can be used as solutions [45]. Thus, in general, only two of the categories above (i.e., workforce flexibility and subcontracting) can be used for the creation of short-time volume flexibility in healthcare industries. Tools that create workforce flexibility usually require the cross-training of staff for several tasks [44]. This is particularly true for systems with substantially high task heterogeneity [14].

Volume flexibility strategies can either be based on internal or external resources, processes, and abilities. Overtime, cross-trained staff, and slack capacity are examples of internal resources, while external staff agencies, outsourcing, and subcontracting are examples of external resources. According to Powers and Jack [15], academic medical centers primarily rely on internal volume flexible strategies which will influence the potential for volume flexibility. The authors also recognize an unwillingness to rely on external resources because of the lack of control and negative impact on the quality of care. 

In this review of the literature, we have found seven tools commonly used for short-term flexibility in healthcare capacity management: the use of overtime, calling in temporary staff, moving staff between units, using internal staffing pools, using external staffing pools, queuing patients, and purchasing care from external providers (subcontractors). These sources are often used as reactive ad hoc solutions to fill in gaps in staff schedules [20,21].

### 2.1. The Use of Overtime

According to several studies, one option for handling a shortage of staff is to use costly overtime solutions [13,21,47,48,49]. Overtime is time spent working beyond employees’ regular working time, and is often divided into two main types: prescheduled fixed overtime and unscheduled overtime [21,50,51]. There are examples from different types of organizations in the literature for the healthcare sector, such as hospitals [47], primary healthcare practices [52], and surgical procedure centers [49]. Overtime for permanent staff (often on an ad hoc basis) is a significant solution for filling in gaps in staff schedules [20,21]. This tool used for short-term volume flexibility is close at hand, because the required staff are already available at the unit when capacity is lacking (i.e., the extra work hours of permanent staff beyond the scheduled end of their shift). Thus, overtime is an easy-to-use tool for workforce flexibility in response to unforeseen events such as sick leave and unanticipated high demand at the point of impact [14,51]. 

However, overtime work can be relatively expensive due to its noticeably higher pay rate compared to regular working hours [14]. In the long term, overtime can have negative consequences for the workforce, since there is a limit to the extent to which employees can work beyond what is initially agreed upon [53]. Accordingly, an increasing workload due to excessive overtime and undesired work schedules has become a primary reason for job dissatisfaction and burnout among nurses [21,54]. 

### 2.2. Calling in Temporary Staff Using Calling Lists

On-call temporary staff, such as part-time nurses and per diem staff, are frequently used in healthcare organizations to respond to variability in demand [20,45,48,53,55]. For example, temporary staff can be nurses that were previously employed at a specific unit and have been granted a pension, but still want to work extra hours. This solution is similar to the use of overtime; it allows for flexibility in the allocation of employees that are already part of the workforce and are required on short notice, although it is a less costly strategy, since overtime fees are not disbursed [55].

### 2.3. Moving Staff Between Units

Jack and Powers [13] found that a tool that can be used to cope with scarce capacity is to relocate staff, during their regular shift or during overtime work, between any units where they are qualified to work. This usually means that some employees are cross-trained to work in more than one unit, and can therefore be reassigned to units where and when they are most needed with short notice [14,56]. According to Qin et al. [14], cross-training is a tool used to gain multiskilled staff suitable for relatively predictable, frequent, and uncertain but stationary conditions (e.g., it is beneficial in situations of absenteeism and demand uncertainty). According to Easton [57], the use of cross-training as a tool to create short-term volume flexibility provides an opportunity to enhance the service level, given the available capacity. However, the benefit of this tool can be offset by inefficiencies such as time wastage when staff move between different units [44], and it may be unfeasible if the involved units have similar demand patterns [58]. Furthermore, cross-training is associated with costs such as training or orientation programs, pool staff support, and wage incentives [22,31,34]. However, in many cases, implementing appropriate orientation programs and pool staff support may be difficult due to resource shortages [34]. Nonetheless, cross-training and other types of flexibility for work assignment present a trade-off rather than an improvement to healthcare capacity management. Ignoring this trade-off will result in an overestimation of the benefits of cross training [59].

Cross-trained staff usually have a home unit where they are stationed primarily and only move to another unit under particular circumstances. This strategy is less costly compared to the use of overtime, and allows for improved quality of patient care compared to the use of an external agency or temporary staff. However, cross-training requires that one or several employees be appropriately trained and continuously updated on the various routines and procedures carried out at different clinics, so that they are able to move between units within a short time frame [14,56,60]. 

As different task types require different types of skills, there are often restrictions on what types of tasks an individual employee can perform [61]; the more specialized a task type, the fewer employees that can be trained to cover it at a reasonable cost or level of productivity [44,61]. However, Pinker and Shumsky [62] show that low utilization in a completely specialized system can decrease service quality and productivity. Hence, their conclusion is that the optimal staff mix combines cross-trained and specialized staff (see [44] for a more comprehensive discussion of different cross-training policies). Karuppan [63] shows that too much cross training may not be good for complex tasks. Therefore, due to learning and forgetting effects [64,65], changes in unit layout, protocols, and support systems in float units [25], cross-trained staff are frequently not as effective as specialists [66].

### 2.4. Using Internal Staffing Pools

A capacity pool is a general capacity source that can be allocated to those parts of the system where the existing workload and capacity demand is unusually high [27,67,68]. Capacity pools are a well-known and extensively used method for improving capacity utilization and the service level in manufacturing firms and service organizations [14,26,69].

Internal staffing pools can be used as a strategy to create short-term flexibility in healthcare organizations [30,31,33], with several examples of this cited in the literature [31,33,42,43]. The examples found in the literature are situated in hospital organizations, although there are no implications that suggest that internal staffing pools could not be used in, for example, primary care centers. 

The use of internal staff pools is similar to moving staff between units, with the main difference being that employees in an internal staff pool are not assigned to a specific home unit, but rather, to the pool itself. Pool staff can be allocated to any unit where they are qualified to fill in gaps in staff schedules on a daily basis. 

### 2.5. Using External Staffing Pools

The use of agency staff can be an effective tool to create short-term volume flexibility [69]. Agency staff are often obtained based on an ad hoc contract that stipulates their role as dealing with short-term variations in capacity, such as absenteeism and turnover, or unforeseen high demand [14]. The use of temporary agency staff is a widespread and increasingly used tool to cope with shortages of staff in healthcare organizations. The costs incurred by Swedish regions for temporary agency staff increased from SEK 1.9 billion in 2010 to 5.6 billion in 2019. The increasing cost of temporary agency staff is not a uniquely Swedish phenomenon. For example, in the United States (US), the cost of temporary agency staff has increased to such an extent that it created financial problems in the sector [25,32,34]. Approximately 75% of US hospitals use staffing agencies as a short-term strategy to resolve staff shortages and to create flexibility in staffing planning [30]. In addition, several studies indicate difficulties, besides the financial issue, in using temporary agency staff in healthcare organizations. These difficulties include impaired patient safety, lower organizational loyalty among external staff compared to permanent staff, a deteriorating work environment, less productive wards, and uncertainty in the supply of agency staff [4,25,30,31,70]. Temporary agency staff are also typically not as productive as internal staff [50].

A measure to reduce the cost of external agency staff is to replace agency staff with less costly internal staffing agencies that provide cross-trained staff to maintain the flexibility that such capacity pools create in staff scheduling [25,30,31,32,33,34]. For example, Region Västra Götaland in Sweden has established a region-wide internal staffing pool to reduce the need to hire costly temporary agency staff. 

### 2.6. Queueing Patients

Queueing patients can be done in both planned and acute care. In planned care, the patient is placed on a waiting list, while in acute care, the queueing of patients result in the patient having to physically wait for care at the emergency department. The number of patients on waiting lists increases when healthcare demand is higher than available capacity [71]. This issue is present in all types of healthcare organizations, and is an increasing problem in Sweden [72]. There are several examples of this in the literature, including surgical units where routine patients are queued due to a lack of resources [52,73,74]. One issue with this solution is that, compared to other short-term solutions to create flexibility in capacity management, there is a risk that patients will not be able to access healthcare within the statutory time frame. In the worst-case scenario, queuing patients can lead to fatal consequences when patients do not receive vital treatment in a timely manner [4,23,75]. 

### 2.7. Purchasing Care from External Providers

A short-term flexibility solution commonly used by Swedish healthcare organizations is to purchase care from external providers when there is a lack of internal capacity [76,77,78]. According to Kumar et al. [23], strategic alliances between healthcare providers result in flexible operations and reduced variability in healthcare delivery. They further argue that rural hospitals depend on urban hospitals for specialty services, which is also the case for Swedish healthcare organizations, where some healthcare supply is allocated to a few centers in the rural country. According to Jack and Powers [45], subcontracting of healthcare services is a frequently used tool to absorb volume fluctuations, particularly in rural hospitals. Moreover, Jack and Powers [37] found that academic medical centers are reluctant to outsource volumes to external sources. 

### 2.8. Summary of the Literature Review

In summary, we have found seven tools used to create volume flexibility in capacity management discussed in the literature: the use of overtime, calling in temporary staff using calling lists, moving staff between units, using internal staffing pools, using external staffing pools, queuing patients, and purchasing care from external providers. These different tools are suitable under different prerequisites and have varying implications on, for example, the work environment, cost, and quality of care. The use of overtime is a costly solution with a long-term negative impact on the work environment, although the use of readily available staff with the requisite knowledge can have temporary benefits. Calling in temporary staff using calling lists is similar to the use of overtime but is not as costly a solution. Moving staff between units requires cross-trained staff and orientation programs, which can be difficult to implement due to resource shortages, but allows for improved quality of care compared to the use of external temporary agency staff. According to Qin et al. [14], cross-training entails substantial prior planning and training, whereas other tools for short-term volume flexibility, such as overtime and on-call temporary staff on calling lists, could be implemented effectively much closer to their eventual use. Using internal staffing pools is similar to moving staff between units; the employees in a capacity pool are not stationed at a specific unit, and therefore, their place of assignment is more flexible. The use of external agency staff is costly and can result in reduced patient safety, a deteriorating work environment, and less productive healthcare units. Queuing patients can result in patient safety issues and, in worst-case scenarios, fatal outcomes for the patients. Finally, purchasing care from external providers allows for flexibility, but academic medical centers are more reluctant to use this tool. 

## 3. Materials and methods

### 3.1. The Setting

Region Västra Götaland delivers care to approximately 1.7 million inhabitants, thus accounting for 17% of Sweden’s population [79]. The region consists of 12 individual hospitals, including four university, eight rural, and four stand-alone hospitals. There are also 202 primary health centers and 28 emergency centers in the region. In addition, there are four private hospitals that have a contractual agreement (i.e., subcontractors) with the healthcare provider in the region. Furthermore, there are capacity pools linked to specific parts of the healthcare system in the region, such as primary care and single hospitals. 

Sahlgrenska University Hospital includes four individual hospitals, and is also the biggest university hospital in Sweden, with 50 specialties. It covers all specialties in the region and accounts for approximately 50% of the total healthcare costs in the region. The hospital has approximately 16,500 employees and 2000 beds. It has 50 specialty departments, including cardiology, clinical physiology, children’s medicine, and psychiatry. A designated manager heads each specialty department; the specialty department managers take overall responsibility for the departments’ capacity planning. Sahlgrenska University Hospital also has an internal staffing pool of mainly nurses and assistant nurses.

Different healthcare units treat different patient groups with varying needs, and therefore, have a diverse set of prerequisites for short-term flexibility in healthcare capacity management [23]. For example, primary care centers can book patients onto waiting lists (i.e., queueing patients) when the demand for healthcare is high, while emergency departments usually cannot reject patients when they appear in the emergency room. Hence, the differences between clinics affect the tools that can be used to achieve short-term flexibility in healthcare capacity management, and therefore, it is necessary to distinguish between different units. Healthcare units can be differentiated according to four levels, which, in turn, results in the division of units into different clusters depending on the type of care:

- Specialty: (1) surgical, (2) medical, or (3) psychiatric care. Surgical patients usually require some kind of surgical procedure, while medical patients require medical management that does not need surgical input. Psychiatric patients suffer from mental, emotional, and/or behavioral disorders that require psychiatric help. In Sweden, the classification of specialties is controlled by Socialstyrelsen [80].
- Admission: (1) inpatient or (2) outpatient care. Inpatient care usually requires admission to a hospital ward, while outpatient care can be delivered at, for example, primary care centers or hospital receptions. In Sweden, the definition of inpatient and outpatient care is outlined in the Swedish Health and Medical Service Act [81].
- Level of urgency: (1) acute or (2) planned care. Acute patients require immediate attention, usually within 24 h, while planned care can be handled over a longer time frame [82].
- Type of organization: (1) primary care center, (2) rural hospital, or (3) university hospital. Primary care centers are responsible for the population’s basic need for care and treatment, regardless of their age or illness. Hospitals deliver specialized care that cannot be supplied in primary care centers. The primary difference between rural and university hospitals is that university hospitals are responsible for education and usually conduct more extensive research compared to rural hospitals [82].

### 3.2. Design and Data Collection

**Figure 1:**
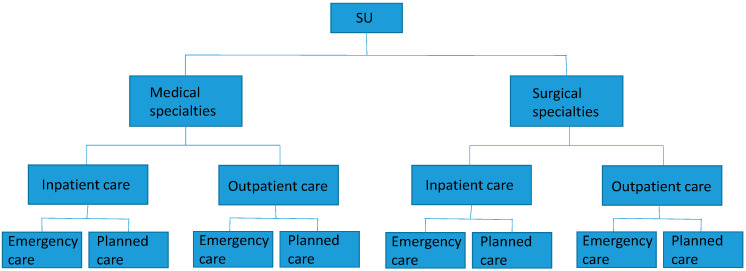
Eight types of specialty departments.

The research was conducted in two stages. First, a prestudy was conducted in the spring of 2018 with personal interviews with ten specialty department managers at Sahlgrenska University Hospital who are responsible for different types of staff, to determine the relevant tools for volume flexibility. To ensure sample representativeness in this study, specialty departments at the hospital were first preclassified dichotomously according to three different dimensions: 1) acute or planned activities, 2) inpatient or outpatient activities, and 3) medical or surgical activities. The ten respondents were chosen from a total of 50 specialty department managers so that all eight possible combinations of dimensions, presented in Figure 1 below, would be covered. For example, combination one includes mainly medical specialties with mainly inpatient and acute activities.

Moreover, department managers at primary health centers in the region were added to cover the local aspect of the healthcare system. Among other things, the respondents described the tools they use for short-term flexibility in capacity management. All interviews were recorded, transcribed, and used as the basis for the data analysis through qualitative content analysis [83]. Second, a survey was conducted to validate the results of the interviews. Based on the results from the interviews and together with the literature review, seven different tools were defined and selected for the questionnaire:

1. Overtime;
2. Temporary staff from internal phone lists;
3. Permanent staff moving across units;
4. Internal staffing pools;
5. External staffing agencies;
6. Queuing patients;
7. Purchase care from external healthcare providers.

A web-based questionnaire was developed with questions regarding the extent to which they used the seven different tools for short-term capacity management. The seven tools were presented to the respondents, and a seven-point Likert scale was used to record answers for each tool, where a lower value indicated a lower level of usage. General questions regarding whether the unit has mainly acute/planned care, inpatient/outpatient care, or surgical/medical/psychiatric care were also asked. The questionnaire also provided open questions to enable the managers to comment on the questions.

The department managers did not emphasize any difference on how short-term capacity management is handled depending on types of profession in the interviews. Furthermore, the literature in the area makes few or no distinctions on how the use of short-term tools differs between types of staff. Hence, the questionnaire was sent to healthcare managers who are responsible for different types of professions, i.e., both unit managers who are responsible of one type of profession (e.g., physicians or nurses) and department managers who are responsible for several types of professions. The questionnaire was tested on the interviewees in the interview study before distribution, as well as on ten managers who were responsible for different types of staff; after minor adjustments, it was sent to 1144 healthcare managers in Region Västra Götaland in the spring of 2019. It should be noted that the questionnaire focused on short-term capacity management during normal circumstances, and not volume flexibility in a system during shock or great pressure. The questionnaire had a response rate of 41.3%, and the distribution of specialties and manager types represented by the participating respondents was in line with the distribution of specialties and manager types in Region Västra Götaland. Hence, we proceeded under the assumption that the data were not characterized by nonresponse bias. A description of the final sample in the study is presented in Table 1 below.

### 3.3. Data Analysis

The general regression model used in the analysis of the survey data is as follows:

(1) $$y_{t}=\beta_{0}+\beta_{1}ACUTE+\beta_{2}OUT+\beta_{3}SUR+\beta_{4}MED+\beta_{5}RUR+\beta_{6}UNI+\varepsilon ,$$

where

- $y_{t}$ is the respondent’s estimated value for tool item t;
- *ACUTE* is the percentage of acute care (in contrast to scheduled care) at the unit where the respondent works;
- *OUT* is the percentage of outpatient care (in contrast to inpatient care) at the unit where the respondent works;
- *SUR* is a binary variable indicating whether the respondent works at a unit that primarily deals with surgical care (= 1) or primarily medical or psychiatric care (= 0);
- *MED* is a binary variable indicating whether the respondent works at a unit that primarily deals with medical care (= 1) or primarily surgical or psychiatric care (= 0);
- *RUR* is a binary variable indicating whether the respondent works at a rural hospital (= 1) or at a university hospital or in primary care (= 0);
- *UNI* is a binary variable indicating whether the respondent works at a university hospital (= 1) or at a rural hospital or in primary care (= 0).

Note that the respondent works at a unit that primarily deals with psychiatric care if SUR = MED = 0, and the respondent works in the primary care if RUR = UNI = 0.

## 4. Results

Table 2 displays the descriptive statistics for the seven tools. As indicated by the mean values, some tools are used to a larger extent than others. A repeated measures ANOVA rejected the null hypothesis that all groups had the same mean value (*F* = 79.5, *p* < 0.001).

Seven different regression models were run to evaluate how the seven tools were used in different healthcare settings. A summary of the results is displayed in Table 3. The detailed tables for the individual regressions appear in Appendix A. The variance inflation factor (VIF) values were generally low, indicating that multicollinearity was not an issue.

The results from the regressions can be summarized as follows:

- The use of overtime is significant and positively correlated with a larger proportion of acute care. Further, overtime is used to a significantly larger extent in medical and surgical units, and it is significantly more common in university hospitals.
- Calling in temporary staff from internal calling lists is significant and positively correlated with a larger proportion of acute care, but significant and negatively correlated with a larger proportion of outpatient care. This tool is also used to a significantly larger extent in surgical units.
- Moving staff between units is significantly more common at rural and university hospitals.
- The use of internal staffing pools is significant and positively correlated with a larger proportion of acute care, but significant and negatively correlated with a larger proportion of outpatient care. Such pools are also used to a significantly lesser extent in medical and surgical units.
- External staffing agencies are used to a significantly lesser extent at university hospitals.
- Queuing patients is a tool that is significant and negatively correlated with a larger proportion of acute care, but it is a significantly more common strategy at surgical units.
- Purchasing care from external providers is significant and negatively correlated with a larger proportion of acute care, and significantly more common in surgical units, rural hospitals and university hospitals.

## 5. Discussion

In our study, we found that the use of overtime is more common in healthcare organizations that mainly provide acute care. This finding is not surprising; the nature of this type of care implies that healthcare capacity is required within a short time frame, and in acute situations, other short-term staffing solutions might not be possible. Moreover, we found that the use of overtime is significantly more common in university hospitals than in primary care centers and rural hospitals, although in our literature review, we found that overtime is used in all kinds of healthcare organizations [47,49,52]. This result might be due to three reasons. First, primary care centers usually have shorter bookings of patients, where each appointment usually lasts for approximately half an hour, while hospitals can have patients admitted to hospital wards for several days up to weeks. Planning capacity for shorter bookings ought to be an easier task than planning capacity for hospital admissions with larger variations in arrival rate and length of stay, which explains why overtime might be used less in primary care centers. Second, primary care centers usually receive patients only during the daytime from Monday to Friday, while hospitals are usually open for admissions during both the day and night, on weekdays, and on weekends. Hence, if patients are admitted to hospital wards and capacity is lacking, the easiest solution might be to use overtime, while primary care centers have both the time and the prerequisites to find other short-term staffing solutions. Thirdly, the difference between rural hospitals and university hospitals might be explained by the degree of specialization. At a university hospital, the staff are, to a higher degree, niched in their expertise, and can therefore be more difficult to replace within a short time frame.

Although overtime is a flexible solution to staff shortages and can improve service delivery [13,48], the solution is costly and there is a practical limit to the extent to which that it can be used and accepted within a workforce [47]. According to Sebastiano et al. [57], the extensive use of overtime can result in poorer job satisfaction and burnout among staff. In our prestudy, several interviewees expressed unwillingness to apply overtime for these reasons. They found it stressful for the staff to always be prepared to work overtime shifts which can cause employees to resign. Furthermore, increasing the workload of staff on duty as scheduled may result in a poorer quality level due to a deteriorating work environment and reduced patient safety [25,30,31,69]. Thus, the use of overtime should be limited. It is important to find alternative solutions for healthcare units that are applying overtime significantly more often, including, for example, the use of capacity pools. 

As found in our literature review, the use of temporary on-call staff is a similar solution to using overtime because it allows for flexibility in the allocation of employees who are already part of the workforce and are required within short notice [55]. Hence, it is not surprising that we found that healthcare units that provide acute care more commonly use temporary staff from phone lists, as well as overtime. Another finding was that the use of temporary on-call staff from phone lists was negatively correlated with there being a larger proportion of outpatient care. One explanation for this might be that inpatient facilities provide healthcare throughout the day, which requires staff to be present at all times, while outpatient clinics have limited opening hours and can redirect patients to other healthcare providers instead of using costly short-term capacity solutions. Finally, we found that the use of temporary on-call staff using phone lists is more common in surgical units. This finding might be a result of our sample; the surgical units might have better access to former employees who are willing to work extra hours compared to other clinics in our sample. For example, one manager stated the following in the questionnaire: “we do not have any temporary staff to call”.

Using temporary staff is a less costly solution compared to overtime [55], but requires access to part-time or per diem employees who are continuously updated on new routines and procedures. For example, several respondents claimed in our questionnaire that they use retired staff and students in their calling lists. Furthermore, according to Sebastiano et al. [53], the use of temporary part-time employees should be preferred over the use of overtime, since the former reduces the risk of fatigue and decreased job satisfaction among employees, which leads to a more stable workforce and reduced costs in the long term. This has implications on units with a higher degree of overtime use, such as university hospitals, and units with a larger amount of acute and somatic care, and should be investigated as an alternative to the use of overtime.

In our study, we found that moving permanent staff between units is significantly more common in rural and university hospitals than in primary care centers. One reason for this might be that hospital units usually have one or more hospital wards located close by, often within the same building, which makes it more feasible to reallocate staff within a short time frame. Primary care centers are usually more isolated, both organizationally and geographically, and hence, it is more complex to move staff between units. According to Qin et al. [14], cross-training can result in benefits such as lower labor costs, higher quality, and increased production flexibility. In addition, according to Inman et al. [56], cross-training can also improve nurses’ morale and job satisfaction. However, this requires that one or several employees are cross-trained and continuously updated on new routines and procedures so that they are able to move between units within a short time frame. The flexibility that could result from cross-training policies might be costly (e.g., education expenses and lost productivity during training) and difficult to maintain, especially concerning complex tasks [44,56,84]. Cross-trained staff may not have the opportunity to rotate frequently enough and practice their additional skills, which creates a nonproductive cycle of training, forgetting, and retraining [56,63], and this could ultimately affect the quality of service delivered [62]. According to Qin et al. [14], cross-trained staff may therefore have lower levels of productivity than specialized staff. Hence, cross-training can be an ambitious task in order to be efficient. However, hospital departments often consist of several units that provide similar types of care, which therefore enables rotating staff between units without extensive cross-training efforts. This might be another reason for why we found that moving permanent staff between units is significantly more common in rural and university hospitals. 

Internal staffing pools can be used to manage variations in the system and reduce workload when demand is unusually high, which can, in turn, lead to an improved work environment [9,27,28,84]. In our study, we found that the use of internal staffing pools is significantly correlated with a larger proportion of acute care. One reason for this finding might be that if resources are scarce in several wards simultaneously, internal staffing pools could be directed from top management to prioritize wards with mainly acute care. For example, one manager answered in the questionnaire that “internal staffing pools are allocated to prioritized units, and unfortunately my unit is not prioritized”. We further found that the use of internal staffing pools is negatively correlated with a larger proportion of outpatient care, and the results of our study indicate that internal staffing pools are used in medical and surgical units to a lesser extent. These findings might be due to the fact that several clinics in our sample are currently not connected to internal staffing pools. For example, one manager stated in the questionnaire, “internal staffing pools are not available for ‘my’ profession but would be a really good idea”. Moreover, medical and surgical units might prefer other short-term staffing solutions, overusing internal capacity pools for several reasons that are not revealed by the questionnaire. One manager answered, “we often contact the internal staffing pool but rarely get help from them”. The use of internal staffing pools is a measure to reduce the use and cost of overtime [30,31,47].

External staffing pools are costly solutions to manage variations in healthcare systems. As stated above, all 20 regions in Sweden operate in accordance with an agreement within Sveriges Kommuner och Landsting (SKL), aiming to become independent of external agency staff in the healthcare sector. This agreement most likely affects the results of our study, since all departments in Region Västra Götaland have been actively working towards reducing the amount of external staffing solutions used. In our study, we found that external staffing agencies are used to a significantly lesser extent at university hospitals. One explanation for this might be that external staffing agencies have shortcomings when it comes to these areas at university hospitals, which often produce highly specialized care, and hence, are used less frequently. Not only is the use of external staffing agencies a costly solution; several studies indicate other problems in addition to the financial cost when using temporary agency staff in healthcare facilities [25,30,31,69].

Queues will increase when healthcare demand and capacity are not balanced [71]. In our study, we found that queuing patients is a tool that is significant and correlates negatively with a larger proportion of acute care, which is an expected result due to the nature of urgent care. Moreover, we found that queuing patients is significantly more common in surgical wards. One reason that surgical wards are overrepresented in these circumstances could be that the surgical process is often complex with multiple actors involved, and when there is a lack of capacity in just one part of the chain, the surgery must be canceled, and therefore, patients will be added to the waiting list. There are several examples in our literature review of surgical units where routine patients are queued due to a lack of resources [52,73,74].

In our study, purchasing care from external providers was found to be negatively correlated with a larger amount of acute care. This is not surprising since it is often planned care that is outsourced to private clinics. Purchasing care from external providers was also more common in rural and university hospitals; according to Kumar et al. [23], rural hospitals often depend on urban hospitals for specialty services. Moreover, strategic alliances between healthcare providers result in flexible operations and reduced variability in healthcare delivery [23]. Although we found that purchasing care is significantly more common in university hospitals compared to primary care centers, Jack and Powers [37] found that academic medical centers are reluctant to outsource volumes to external sources. In Sweden, the Health and Medical Service Act regulates the maximum allowed waiting times from seeking healthcare, to first visit and surgery or treatment. Hence, although university hospitals could be reluctant to purchase care from external providers, as Jack and Powers [37] argue, they might not have any feasible alternatives in order to follow Swedish laws and regulations. Another finding from the study was that surgical units more commonly purchase healthcare from external providers. Shorter surgical inventions are quite convenient to outsource, since they usually result in a limited length of stay and are often one-time interventions for the patient.

## 6. Conclusions

In this study, we have found where and to what extent the major types of tools for short-term flexibility are used among healthcare managers in a regional healthcare system in Sweden. This study, while being limited to a Swedish context, has several managerial implications for healthcare providers, including finding alternative flexibility solutions for the types of units where costly, inefficient, and low-quality solutions are often applied. Units that frequently apply reactive flexibility solutions such as the use of overtime would benefit from moving towards the proactive use of short-term capacity allocation, such as using cross-trained personnel or taking a capacity pooling approach. For example, units that frequently apply overtime or queuing patients as flexibility solutions when capacity is lacking would benefit from a long-term perspective if alternative tools could be applied in the operational allocation of resources. Moreover, different types of units have different prerequisites for the short-term allocation of resources. For example, primary care centers can apply solutions that do not require immediate reallocation of capacity more often, such as queuing patients. However, queuing patients, from a patient safety perspective, is inefficient and undesirable, which is why alternative proactive solutions should be found and implemented. 

The results have implications for future research on how capacity pools can be organized and implemented for different healthcare units to increase operational flexibility and use resources more efficiently. Units that frequently use short-term flexibility tools that have a negative impact on cost, work environment, and quality of care, such as the use of overtime, external staffing agencies, or queuing patients, must find alternative short-term staffing solutions. These reactive flexibility solutions often lead to the inefficient use of resources and long-term difficulties in matching available capacity to demand. The results can be used to determine how a proactive capacity pooling approach can be used and implemented to, for example, identify the types of units that would benefit the most from such an approach. Furthermore, the study focused on short-term staffing solutions during normal circumstances. An interesting area for future research would be to investigate the creation of volume flexibility in a system during shock or great pressure due to, for example, pandemics.

## Figures and Tables

**Table 1:** Descriptive statistics of the sample.

Parameter	Number of Respondents
**Specialty**	
Surgical	98
Medical	285
Psychiatric	75
Other	15
**Admission**	
Mainly inpatient care	236
Mainly outpatient care	237
**Level of urgency**	
Mainly acute care	211
Mainly planned care	262
**Type of organization**	
Primary care center	75
Rural hospital	216
University hospital	182

**Table 2:** Descriptive statistics for the seven tools.

Tool	Mean	S.D.	95% Confidence Interval	
			Lower Bound	Upper Bound
1. Using overtime	4.29	1.94	4.11	4.47
2. Calling in temporary staff from “internal phone lists”	4.25	2.25	4.03	4.46
3. Moving staff between units	4.03	2.02	3.84	4.23
4. Using internal staffing pools	3.03	2.26	2.80	3.26
5. Using external staffing agencies	1.87	1.70	1.70	2.05
6. Queueing patients	3.30	2.22	3.07	3.54
7. Purchasing care from external providers	2.16	1.80	1.96	2.36

**Table 3:** Results (standardized beta values) from the regressions.

	Model						
Variable	1	2	3	4	5	6	7
ACUTE	0.283 ***	0.377 ***	0.041	0.285 ***	0.025	−0.314 ***	−0.154 **
OUT	−0.083	−0.104 *	−0.077	−0.14 **	−0.047	0.113	0.06
SUR	0.26 ***	0.203 **	0.055	−0.141 *	0.022	0.185 **	0.341 ***
MED	0.23 ***	0.04	−0.003	−0.278 ***	0.051	0.03	0.067
RUR	0.107	−0.035	0.253 **	−0.075	−0.052	0.064	0.348 ***
UNI	0.16 *	−0.106	0.361 ***	0.02	−0.287 **	−0.042	0.262 **
R^2	0.183	0.207	0.096	0.168	0.064	0.15	0.15
F	16.14 ***	17.31 ***	7.35 ***	12.11 ***	4.03 **	9.51 ***	9.08 ***

Note: *** *p* < 0.001. ** *p* < 0.01. * *p* < 0.05.

## Appendix A

The tables below show the individual regressions.

**Table A1:** Regression results: Using overtime.

	B	S.E.	Std.Beta	*t*	*p*-Value	*F*-Value	R^2^
(Constant)	2.557	0.359		7.119	<0.001	16.1 (*p* < 0.001)	0.183
ACUTE	0.015	0.002	0.283	5.990	<0.001		
OUT	−0.004	0.002	−0.083	−1.698	0.090		
SUR	1.242	0.269	0.260	4.620	<0.001		
MED	0.910	0.228	0.230	4.000	<0.001		
RUR	0.415	0.273	0.107	1.520	0.129		
UNI	0.637	0.279	0.160	2.286	0.023		

**Table A2:** Regression results: Calling in temporary staff from “internal phone lists”.

	B	S.E.	Std.Beta	*t*	*p*-Value	*F*-Value	R^2^
(Constant)	3.353	0.436		7.689	<0.001	17.3 (*p* < 0.001)	0.207
ACUTE	0.023	0.003	0.377	7.788	<0.001		
OUT	−0.005	0.003	−0.104	−2.064	0.040		
SUR	1.118	0.320	0.203	3.496	0.001		
MED	0.184	0.272	0.040	0.678	0.498		
RUR	−0.159	0.334	−0.035	−0.477	0.633		
UNI	−0.492	0.346	−0.106	−1.423	0.155		

**Table A3:** Regression results: Moving staff between units.

	B	S.E.	Std.Beta	*t*	*p*-Value	*F*-Value	R^2^
(Constant)	3.008	0.401		7.496	<0.001	7.3 (*p* < 0.001)	0.096
ACUTE	0.002	0.003	0.041	0.808	0.420		
OUT	−0.004	0.002	−0.077	−1.479	0.140		
SUR	0.273	0.301	0.055	0.909	0.364		
MED	−0.014	0.255	−0.003	−0.055	0.956		
RUR	1.021	0.312	0.253	3.279	0.001		
UNI	1.487	0.317	0.361	4.696	<0.001		

**Table A4:** Regression results: Using internal staffing pools.

	B	S.E.	Std.Beta	*t*	*p*-Value	*F*-Value	R^2^
(Constant)	3.581	0.485		7.379	<0.001	12.1 (*p* < 0.001)	0.168
ACUTE	0.018	0.003	0.285	5.443	<0.001		
OUT	−0.007	0.003	−0.140	−2.618	0.009		
SUR	−0.769	0.342	−0.141	−2.247	0.025		
MED	−1.273	0.295	−0.278	−4.311	<0.001		
RUR	−0.336	0.382	−0.075	−0.881	0.379		
UNI	0.094	0.391	0.020	0.240	0.810		

**Table A5:** Regression results: Using external staffing agencies.

	B	S.E.	Std.Beta	*t*	*p*-Value	*F*-Value	R^2^
(Constant)	2.251	0.385		5.85	<0.001	4 (*p* = 0.001)	0.064
ACUTE	0.001	0.003	0.025	0.441	0.659		
OUT	−0.002	0.002	−0.047	−0.803	0.423		
SUR	0.091	0.281	0.022	0.322	0.747		
MED	0.178	0.239	0.051	0.745	0.457		
RUR	−0.178	0.288	−0.052	−0.617	0.538		
UNI	−1.001	0.298	−0.287	−3.365	0.001		

**Table A6:** Regression results: Queueing patients.

	B	S.E.	Std.Beta	*t*	*p*-Value	*F*-Value	R^2^
(Constant)	3.418	0.494		6.924	<0.001	9.5 (*p* < 0.001)	0.147
ACUTE	−0.020	0.004	−0.314	−5.659	<0.001		
OUT	0.006	0.003	0.113	1.964	0.050		
SUR	0.996	0.361	0.185	2.760	0.006		
MED	0.138	0.308	0.030	0.447	0.655		
RUR	0.285	0.359	0.064	0.795	0.427		
UNI	−0.193	0.360	−0.042	−0.536	0.593		

**Table A7:** Regression results: Purchasing care from external providers.

	B	S.E.	Std.Beta	*t*	*p*-Value	*F*-Value	R^2^
(Constant)	0.918	0.418		2.194	0.029	9.1 (*p* < 0.001)	0.152
ACUTE	−0.008	0.003	−0.154	−2.676	0.008		
OUT	0.003	0.003	0.060	1.018	0.309		
SUR	1.454	0.294	0.341	4.945	<0.001		
MED	0.242	0.254	0.067	0.953	0.341		
RUR	1.257	0.317	0.348	3.966	<0.001		
UNI	0.957	0.319	0.262	3.003	0.003		
